# Blue light optimizes photosynthetic physiology and photoprotective molecular mechanisms of *Hydrangea quercifolia* seedlings

**DOI:** 10.3389/fpls.2026.1951910

**Published:** 2026-10-13

**Authors:** Pengfei Su, Dacheng Wang, Yameng Gao, Xue Chen, Jinyan Hou, Lifang Wu

**Affiliations:** 1 College of Biological and Chemical Engineering, Qilu Institute of Technology, Jinan, China; 2 Institute of Intelligent Machines, Hefei Institutes of Physical Science, Chinese Academy of Sciences, Hefei, China; 3 University of Science and Technology of China, Hefei, China; 4 Institute of Hefei Artificial Intelligence Breeding Accelerator Co Ltd., Hefei, China

**Keywords:** blue light, hormone signaling, *Hydrangea quercifolia*, photosynthetic physiology, seedling growth

## Abstract

**Introduction:**

*Hydrangea quercifolia* is a rare ornamental shrub whose natural populations have declined due to habitat loss, necessitating efficient artificial propagation techniques. Light quality is a critical environmental factor regulating seedling growth and physiological quality; however, the molecular mechanisms by which different LED light spectra modulate photosynthetic physiology and hormone signaling in *H. quercifolia* remain poorly understood.

**Methods:**

We investigated the regulatory mechanisms of four LED light qualities—white (W), blue (B), red (R), and red:blue = 3:1 (R3B1)—on the growth, photosynthetic performance, chloroplast ultrastructure, hormone metabolism, and transcriptomic profiles of *H. quercifolia* 'Harmony' seedlings. Physiological parameters were measured on days 7, 14, and 21, and RNA sequencing was performed to identify differentially expressed genes and associated signaling pathways.

**Results:**

Blue (B) light significantly increased chlorophyll, anthocyanin, and cytokinin (CTK) contents, promoted chloroplast proliferation and grana thylakoid development, and enhanced photosynthetic efficiency. Transcriptomic analysis revealed that B light upregulated genes encoding photosystem I (PSI) and photosystem II (PSII) reaction center proteins (especially *PsbS*, Photosystem II S subunit), strengthened non-photochemical quenching (NPQ) and photoprotective capacity, and reduced brassinosteroid (BR) biosynthesis by suppressing *BRI1* (Brassinosteroid Insensitive 1) and *BSK* (Brassinosteroid-Signaling Kinase) expression. In contrast, R3B1 and red (R) light induced chloroplast shrinkage, reduced chloroplast number, and triggered starch accumulation. R3B1 light inhibited PSII oxygen evolution and electron transport, downregulating chloroplast-related genes; while R light suppressed cytokinin biosynthesis by downregulating *CRE1* (Cytokinin Response 1) and activated *BAK1* (BRI1-Associated Receptor Kinase 1)-mediated BR signaling.

**Discussion:**

These findings demonstrate that blue light plays a pivotal role in promoting the high-quality development of hydrangea seedlings by optimizing photosynthetic apparatus assembly, enhancing photoprotection, and repressing BR-mediated growth inhibition. The differential molecular responses to distinct light spectra provide a theoretical basis for designing optimized artificial light environments and improving the efficiency of horticultural seedling production.

## 1 Introduction

In controlled-environment agriculture, light serves as a promising tool to influence plant growth, development, and the production of secondary metabolites, ultimately enhancing plant quality (Contreras-Avilés et al., 2024). The significance of the light environment lies in the ability of plants to convert light energy into chemical energy through photosynthesis, synthesizing organic compounds, which form the foundation of plant growth and development (Sage, 2004). During photosynthesis, the photopigments chlorophyll a (chl a), chlorophyll b (chl b), and carotenoids show the highest absorption efficiency within blue (B, 315–400 nm) and red light (R, 600–700 nm) regions. Consequently, blue and red light currently represent the most extensively studied spectral regions in plant photobiology. R and far-red light (700–750 nm) are perceived by plants through phytochromes (phyA–phyE), while B is detected by cryptochromes (CRY1, CRY2, CRY3), phototropins (PHOT1, PHOT2), and F-box flavin-binding proteins (Franklin and Quail, 2009). However, when plants are grown under conditions that differ from their natural habitats, both the quantity and quality of their growth and metabolic components may change. This species-specific variability necessitates targeted investigation of individual species when using light environments to regulate growth and development, thus ensuring a reliable basis for optimizing light conditions for a given population.

In recent years, methods for regulating the light environment have continuously evolved. Due to their low energy consumption, high luminous efficiency, and controllable wavelengths, LED light sources are currently the most widely used lighting systems in modern agriculture, including in plant growth chambers on the International Space Station (Mickens et al., 2018). By employing LED-based light regulation, it is possible to precisely design illumination strategies, increase planting density, shorten growth cycles, and markedly improve the efficiency of light energy utilization. Numerous studies have reported the effects of different red-to-blue light ratios on plant growth and development. R light exposure enhances both the antioxidant capacity and primary metabolite content in vegetables, including cucumbers and tomatoes (Guo et al., 2022; Zhang et al., 2024). Compared with other LED light qualities, blue light enhances anthocyanin content in tomato seedlings (Zhang et al., 2024), whereas anthocyanin production in cranberry fruit appears to be most sensitive to R light. In addition, blue light can increase the carotenoid content in coffee and the ascorbic acid content in lettuce and komatsuna, although it does not affect spinach (Yamazaki et al., 2000; Roldán et al., 2008). LED illumination enables plant growth and development to be regulated by altering the spectral composition, thereby offering a reliable approach for further improving the efficiency of plant factory propagation. Many studies have explored how red-blue light environments affect plant growth and metabolism. Regardless, red-blue light environments are expected to further enhance the efficiency of horticultural crop propagation under controlled factory conditions. However, much remains unknown about the mechanisms by which horticultural species respond to LED red-blue light, especially concerning the links between light environment-responsive genes and the contents of metabolites related to photosynthesis and endogenous hormones.

Historically, the fields of photobiology and plant hormone signaling, both critical areas of plant physiology, were typically studied in isolation. Until recently, they have only been frequently examined in combination. By interacting with multiple plant hormones, light plays a central role in regulating transitions between photomorphogenesis and skotomorphogenesis (de Wit et al., 2016). In photomorphogenesis, cytokinins, abscisic acid, and strigolactones act as promoters, while gibberellins (GAs), brassinosteroids (BRs), ethylene, and auxin tend to support skotomorphogenesis (Chen et al., 2008; de Lucas et al., 2008; Feng et al., 2008; Shi et al., 2011; Rasmussen et al., 2012; Yu et al., 2013). Light inhibits GA signaling by reducing GA biosynthesis and enhancing the activity of DELLA proteins (Achard et al., 2007; Alabadí and Blázquez, 2009), Moreover, HY5 further lowers GA levels by upregulating the expression of GA-degrading enzymes such as GA2ox2, thus restricting the transcriptional activity of PIF4 (de Lucas et al., 2008; Feng et al., 2008; Weller et al., 2009). Regarding BRs, light promotes the expression of *MSBP1*, which interacts with *BRI1*, a core BR signaling receptor, to repress BR signaling. Additionally, interference with *BZR1* transcriptional activity and protein stability further weakens the influence of BR signaling (Shi et al., 2011; Li et al., 2015). Cytokinins and strigolactones, on the other hand, stabilize HY5 protein by reducing COP1 accumulation in the nucleus, thereby indirectly promoting photomorphogenesis (Tsuchiya et al., 2010; Jia et al., 2014). Although plant hormone interaction networks have been partially delineated, numerous regulatory pathways remain unresolved, even in Arabidopsis. For example, aside from its known role in stabilizing HY5, the link between cytokinins and this integrator of light signaling remains unclear. Consequently, there are evidently many more unresolved complexities in the regulatory networks governing the interplay of light with growth, development, and metabolism in other plant species.

*Hydrangea quercifolia*, native to the southeastern United States, has drawn the attention of landscape professionals due to its seasonal variations in flower and leaf coloration as well as its superior cold tolerance. Although some regions still maintain relatively high population densities of this species, its native range is diminishing (Sherwood et al., 2022). A recent survey revealed that 23% of previously recorded populations no longer exist, primarily due to habitat loss or degradation, often caused by invasive species or commercial development (Sherwood et al., 2023). Against this backdrop, the selection of high-quality seedlings and factory-scale propagation have become critical factors for sustainable development within the ornamental industry and for ensuring ecological security. Changes in light conditions profoundly influence plant morphogenesis, physiology, and metabolism. Therefore, by investigating the growth patterns of ‘Harmony’ under various light environments, it is possible to design precise illumination strategies, shorten growth cycles, maximize light energy utilization, and achieve efficient biomass accumulation. In earlier studies, our research team established an efficient *in vitro* regeneration system for ‘Harmony’ using LED-based light regulation technologies (Su et al., 2024). These studies indicated that different ratios of LED red and blue light significantly affected photomorphogenesis and secondary metabolism during seedling regeneration, and that hormone levels were strongly influenced by these light conditions. However, the molecular mechanisms underlying the marked differences in photosynthetic characteristics and hormone levels in ‘Harmony’ under different light environments remain to be elucidated.

Based on the identified research gaps and practical industrial demands, this study systematically investigated the regulatory effects of white light, monochromatic blue (B) light, monochromatic red (R) light, and red–blue combined (R3B1) light on seedling growth, photosynthetic physiology, endogenous hormone metabolism, and transcriptomic characteristics of the ornamental hydrangea cultivar ‘Harmony’. The core objective was to elucidate the molecular regulatory network connecting LED light spectra, photosynthetic performance, and hormone signaling in hydrangea seedlings, and to clarify the differential response mechanisms of woody ornamental horticultural plants to varied red and B light conditions. Practically, the results fill the technical gap of precise light regulation for industrial seedling production of hydrangea, providing feasible and optimized light environment strategies for factory-scale propagation, high-quality seedling cultivation, and quality improvement of hydrangea and other woody horticultural crops. In addition, the key light-responsive genes and metabolic pathways identified in this study establish a molecular basis for subsequent genetic modification and precision breeding of ornamental hydrangea, which contributes to the dual enhancement of production efficiency and commercial value in horticultural industrialization. In this study, LED light sources were used to cultivate ‘Harmony’ seedlings. We evaluated the growth and physiological responses of ‘Harmony’ to different light qualities (White, B, R, and R3B1 light), and further explored the underlying molecular mechanisms via Illumina transcriptome sequencing, with a focus on differentially expressed genes involved in photosynthesis and hormone metabolism.

This study established a high-quality reference transcriptome database for ‘Harmony’ and obtained high-quality transcripts. These results lay a foundation for further investigation into the changes and regulatory mechanisms of key genes, as well as photosynthesis and hormone metabolic pathways, under different light spectra. Ultimately, this work provides solid scientific evidence and technical support for large-scale production, quality improvement, and molecular breeding of ‘Harmony’ and other horticultural crops.

## 2 Materials and methods

### 2.1 Plant materials and growing conditions

*Hydrangea quercifolia* ‘Harmony’ was provided by Fuluola Agriculture Co., Ltd. of Henan Province, China. The experimental plant materials were collected from three-year-old plants cultivated at the company’s production base. Healthy and vigorously growing individuals were selected from the field, and young stem segments (approximately 5–10 cm in length) were excised for experimental use. The collected stems were subsequently sterilized and micropropagated using Murashige and Skoog medium, which had been specifically optimized to achieve rapid clonal multiplication. The medium was solidified with 6.8 g L^-1^ agar, a concentration empirically determined to maximize shoot proliferation while maintaining hydration stability. The pH of the medium was adjusted to 5.8 using either 1N NaOH or 1N HCl and subsequently sterilized by autoclaving at 121 °C and 105 kPa for 20 minutes. After sterilization, IAA was added to a final concentration of 0.41 mg L^-1^. The culture vessels consisted of transparent 280 mL tissue culture bottles, each containing approximately 40 mL of the prepared medium.

Seedlings were cultivated under white LED illumination within controlled environmental conditions: temperature of 22 ± 1 °C and relative humidity ranging from 60 % to 70 %. When the third to fourth true leaves of ‘Harmony’ seedlings were fully expanded, seedlings with consistent growth performance were selected for exposure to various light-quality treatments.

Sprouts were cultivated using custom-built lighting equipment equipped with four independent modules to enable simultaneous plant cultivation under separately adjustable light regimes. Commercially available LEDs (Xiamen Rural Hui Photoelectric Technology Co. Ltd.) were utilized to produce red light (660 nm) and blue light (450 nm). The 1.2m-long lamp tubes for sprout illumination had a rated power output of 36 W per tube, with double-row beads of distinct light sources evenly arranged inside each tube. The ratios of red to blue light were defined according to measured spectral composition rather than bead number, owing to discrepancies in luminous efficiency between red and blue LED beads. The photosynthetic photon flux density (PPFD) was maintained at 160 ± 5 μmol m^-^² s^-1^ by adjusting the suspension height of light modules.

Four distinct light-quality treatments were established in this study (**Table 1**): White light served as the control group; two monochromatic-light treatments including R and B light; and one combined red-blue light treatment, referred to as R3B1, in which the red-to-blue ratio was set at 3:1 on a photosynthetic photon flux density (PPFD) basis. The photoperiod was set to 16 h light/8 h dark. A handheld portable spectroradiometer (Spectra Pen LM510, Drasov, Czech Republic) was employed to measure spectral distribution and light intensity (**Figure 1**).

**Table 1:** Incident PPFD for each radiation wavelength in each light treatment.

Light treatment	PPFD (μmol m^-2^ s^-1^) of each wavelength in each light treatment			
	Blue	Intermediate	Red	Total
White	–	–	–	160
R	0	0	160	160
R3B1	40	0	120	160
B	160	0	0	160

PPFD means photosynthetic photon flux density. White means white light. R and B mean red light and blue light. R3B1 indicate the spectral proportion of red and blue light are 3:1.

**Figure 1:**
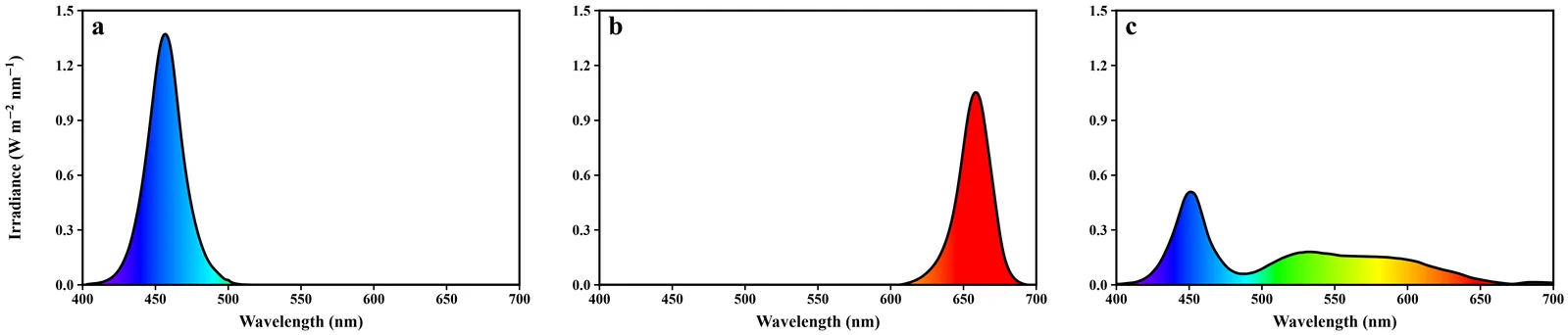
Spectral characteristics of B **(A)**, R **(B)** and White **(C)** light used in the experiments. R3B1 light was obtained by combining B and R light as shown in **Table 1**.

### 2.2 Growth parameters

Twenty-one days after the light treatments, plant height and leaf area were measured. Plant height was determined using a ruler, and leaf area was calculated using ImageJ software. The aboveground parts of ‘Harmony’ were harvested, and the fresh weight (g) of the aboveground portion in each culture bottle was recorded. The aboveground tissues were then placed in breathable paper bags, subjected to blanching at 100 °C for 20 minutes, and subsequently dried in an oven at 80 °C overnight until a constant weight was achieved. The dry weight (g) was then measured, and the dry matter content (%) was calculated. Each treatment had six replicates, with each culture bottle representing one replicate (each containing five explants). All experiments were performed in triplicate. Chlorophyll and anthocyanin content determination.

Chlorophyll extraction was performed using the alcohol-acetone extraction method. A 0.2 g fresh sample of leaves was placed in a centrifuge tube containing a 1:1 (v/v) mixture of ethanol and 80% acetone. The samples were left to stand in the dark for at least 24 hours until the leaves turned White. Subsequently, absorbance (A) was measured at wavelengths of 470 nm, 663 nm, and 645 nm. The concentrations of Chl a and Chl b in the leaves were determined following the chlorophyll determination method outlined by Lichtenthaler and Wellburn (1983), using the following equations:


$$chla\;\left(mg\;g^{-1}\right)\;=\;\left(12.72A663-2.59A645\right)\;\times V\times F/W$$

$$chlb\;\left(mg\;g^{-1}\right)\;=\;\left(22.88A645-4.67A663\right)\times V\times F/W$$

Where: V=Total volume of the extraction solution (mL), F = Dilution factor (1000 in this protocol), W = Fresh weight of the sample (g).

To determine anthocyanin content, the pH differential method was employed, which is based on the changes in absorbance characteristics of anthocyanins under different pH conditions. A 0.2 g fresh sample of leaves was frozen in liquid nitrogen and then crushed into a powder. The powder was added to 10 mL of 60% ethanol solution (containing 96 mM hydrochloric acid) and subjected to ultrasonic crushing at a power of 300 W in an ice-water bath for 20 minutes. Subsequently, the mixture was extracted at 60 °C for 20 minutes. After extraction, the evaporated liquid was supplemented to 10 mL, and the samples were centrifuged at 10,000 × g at room temperature for 10 minutes to obtain the supernatant for measurement. The determination process was conducted according to the method described by Lee et al. (2005).

### 2.3 Measurement of chlorophyll fluorescence OJIP curves

Chlorophyll fluorescence parameters were measured in the third true leaf of ‘Harmony’ grown under White, B, R3B1, and R light conditions on days 7, 14, and 21. A Fluorpen FP110 (Czech Republic) was used for measurements. Prior to testing, the third true leaf was dark-adapted for at least 20 minutes using a leaf clip, and chlorophyll fast fluorescence kinetics (i.e., the OJIP curve) were recorded through pulse amplitude modulation (PAM) technology. In the measurements, Fo corresponds to the fluorescence value at 20 µs, Fk at 300 µs, Fj at 2 ms, Fi at 30 ms, and F_M_ is the maximum fluorescence at the P point. The OJIP curve was normalized to eliminate extraneous factors (e.g., leaf thickness, pigments, and surface attachments) that might affect curve differences:


$$Vt\;=\;\left(F_{t}-Fo\right)/\left(Fp-Fo\right)$$

$$\Delta Vt\;=\left(Vt_{-treatment}-Vt_{-control}\right)$$

Where Ft is the fluorescence value at time t on the OJIP curve, Vt is the variable fluorescence of the normalized OJIP curve, and ΔVt is the relative variable fluorescence between the treatment and control groups.

### 2.4 Chloroplast transmission electron microscopy

After 21 days of growth under White, B, R3B1 and R light conditions, the same region of the third true leaf from seedlings was sampled using a 2 mm diameter punch. For each light treatment, three independent seedling biological replicates were set, and the same fixed region of the third true leaf of healthy and uniformly growing seedlings was selected. The samples were promptly immersed in 2.5% glutaraldehyde fixative pre-cooled to 4 °C. A vacuum treatment was then conducted to facilitate leaf settling, and the samples were sent to Scientific Compass Testing Company for transmission electron microscopy (TEM) sample preparation.

First, the fixative was removed, and the samples were washed with 0.1 M phosphate-buffered saline (PBS) at pH 7.0 for 15 minutes, repeated three times. Subsequently, the samples were treated with 1% osmium tetroxide for 1–2 hours to achieve fixation, followed by three 15-minute washes in PBS. The samples were dehydrated through a graded ethanol series (30%, 50%, 70%, 80%, 90%, and 95%) for 15 minutes each. Finally, they were sequentially treated with 100% ethanol and acetone for 20 minutes each. The samples were then immersed in a mixture of embedding medium and acetone at volume ratios of 1:1 and 3:1 for 1 hour and 3 hours, respectively, and maintained overnight at 70 °C in the embedding medium. Afterward, the samples were sectioned into ultrathin slices (approximately 80 ± 10 nm) and stained with lead citrate [Pb_3_(C_6_H_8_O_7_)_2_] and 50% ethanol-saturated uranyl acetate [UO_2_(CH_3_COO)_2_] solutions for 5–10 minutes each. Once dried, the stained sections were ready for TEM observation. A total of 15 valid TEM images obtained from all treatment groups were systematically analyzed to characterize the differences in leaf and chloroplast ultrastructure under different light quality conditions.

### 2.5 Determination of endogenous hormone content

After 21 days of cultivation under different light quality conditions, the third to fourth true leaves of seedlings exhibited stable physiological status and fully developed photosynthetic function. Therefore, these mature functional leaves were selected as samples to accurately reflect the effects of different light spectra on seedling growth and metabolism and ensure experimental stability and credibility. For each biological replicate, 0.5 g of leaf tissues was collected from seedlings within the same culture vessel. Each light treatment included three independent biological replicates, with no mixing of tissues from different vessels or individual plants. Immediately after sampling, all fresh samples were rapidly frozen in liquid nitrogen. The frozen samples were sent to Shanghai Hengyuan Biotechnology Co., Ltd. for ELISA (Enzyme-Linked Immunosorbent Assay) analysis of GA, cytokinins (CTK), and BR to determine the content of endogenous hormones under varying light conditions.

### 2.6 RNA extraction and RNA-seq analysis

‘Harmony’ seedlings cultivated under different light quality conditions for 21 days were selected. Approximately 0.2 g of experimental material was collected, immediately frozen in liquid nitrogen, and subjected to tissue disruption using a tissue homogenizer. Total RNA was extracted using a plant RNA extraction kit (M5 Quickspin, Beijing Juheymei, China) following the manufacturer’s protocol. The extracted RNA samples were analyzed by 1% agarose gel electrophoresis to assess RNA degradation and potential contamination. The RNA gel electrophoresis analysis of ‘Harmony’ seedlings cultured under different light conditions for 21 days is shown in **Supplementary Figure S1**. RNA purity and concentration were determined using a Scandrop spectrophotometer (Analytikjena, Germany). All collected samples included three independent biological replicates. Sequencing was performed using the PE150 mode on a high-throughput RNA-seq platform (Beijing Baimike Biotechnology Co., Ltd., China). The *Hydrangea quercifolia* genome: *Hydrangea_quercifolia*.Hquercifolia_589_v1.1.genome.fa was used as the reference for sequence alignment and subsequent analysis. The alignment of clean reads to the reference genome was conducted according to the HISAT2 (Kim et al., 2015) protocol, and the assembly of reads was performed following the StringTie software workflow.

### 2.7 Gene expression analysis related to photosynthesis and hormone metabolism

Differential expression genes (DEGs) were identified in ‘Harmony’ seedlings cultivated under different light quality conditions for 21 days. During the DEG detection process, genes with a false discovery rate (FDR) <0.01 were selected as DEGs. Based on the transcriptome sequencing data and genome annotation of Hydrangea, DEGs with larger log_2_ fold changes (log_2_FC) in photosynthesis and hormone metabolism pathways were further screened. Additionally, key metabolic pathways and regulatory genes associated with the target traits were determined through KEGG (Kyoto Encyclopedia of Genes and Genomes) annotation analysis.

### 2.8 Statistical analysis

Data were reported as the mean ± SD. Biochemical indices were determined by three biological repetitions and three technical repetitions. The average value of each test trial was analyzed by one-way ANOVA, and either Dunnett’s T3 multiple comparison test was used to determine significant differences at *P* < 0.05. All analyses were conducted using GraphPad Prism 9 (GraphPad, San Diego, CA, USA). In the figures, ^*^, ^**^, ^***^, and ^****^ indicate significant differences at the 0.05, 0.01, 0.001, and 0.0001 significance levels, respectively, whereas the absence of a label indicates no significance.

Transcriptome data normalization and all statistical analyses were performed using R software. Gene expression levels were uniformly normalized to FPKM (Fragments Per Kilobase of transcript per Million mapped reads) for subsequent multivariate analysis. Principal component analysis (PCA) and Spearman’s correlation analysis were conducted using the ggplot2 and corrplot packages, respectively, to evaluate sample reproducibility and overall transcriptional differences across treatments. Hierarchical clustering analysis was performed on FPKM-normalized expression profiles, with Euclidean distance as the distance metric and Ward’s minimum variance method as the clustering linkage algorithm. DEG screening was performed based on raw gene count values. Specifically, the DESeq2 package was used for groups with biological replicates, while the edgeR package was applied for groups without biological replicates. The unified DEG screening criteria were set as Fold Change≥2 and FDR<0.01. The Benjamini–Hochberg method was employed for multiple-testing correction to control false discovery rate, and log_2_-transformed fold change (log_2_FC) was used to quantify the magnitude of gene expression changes.

## 3 Results

### 3.1 Growth parameters

Different light spectra significantly regulate the growth and development of ‘Harmony’. After 21 days of light treatment, leaf area significantly increased under B light but decreased under R3B1 and R light (**Figure 2A**). Compared to the White light control, plant height and dry matter content showed no significant difference under B light, whereas both parameters decreased significantly under R3B1 and R light (**Figures 2B, C**). Fresh weight increased significantly under B light, remained unchanged under R3B1 light, and decreased significantly under R light (**Figure 2D**). The ‘Harmony’ seedlings cultivated under different light conditions for 21 days are shown in **Figure 2E**.

**Figure 2:**
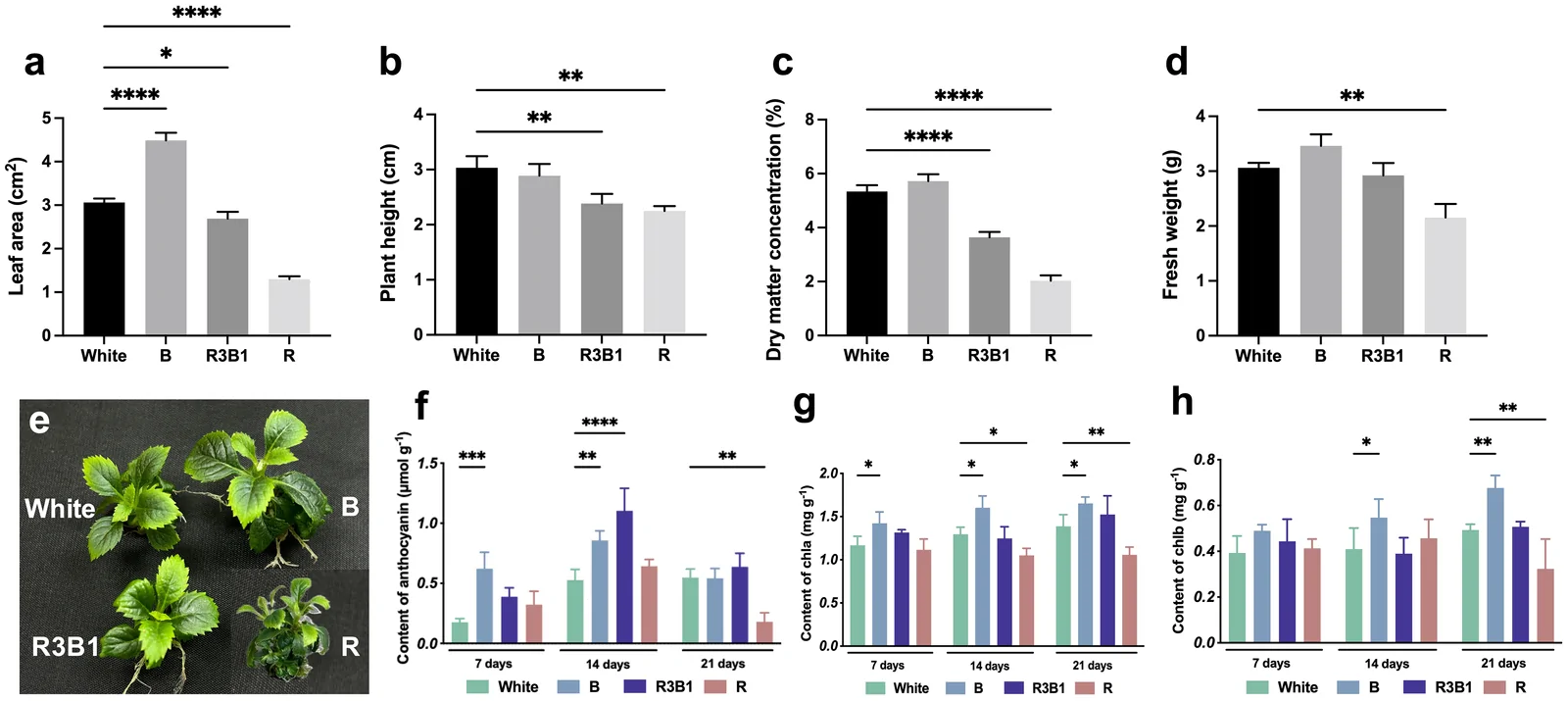
Leaf area **(A)**, plant height **(B)**, dry matter content **(C)**, fresh weight **(D)**, and growth vigor **(E)** of ‘Harmony’ under different light conditions, and the contents of anthocyanins **(F)**, Chl a **(G)**, and Chl b **(H)**. *P < 0.05, **P < 0.01, ***P < 0.001, ****P < 0.0001.

### 3.2 Blue light promoted the biosynthesis of both chlorophyll and anthocyanin

Chlorophyll and anthocyanin accumulation in hydrangea seedlings exhibit obvious light-quality-dependent and time-specific response patterns. Anthocyanin content rose under all light conditions (**Figure 2F**). On day 7, B light resulted in 0.28 ± 0.03 mg g^-1^ of anthocyanin, whereas R generated only 0.15 ± 0.01 mg g^-1^. Over time, R3B1 became more effective at enhancing anthocyanin levels, rising from 0.24 ± 0.02 mg g^-1^ on day 7 to 1.16 ± 0.08 mg g^-1^ on day 14. In the initial 7 days, B light showed the strongest promotion of anthocyanin synthesis. Therefore, the short-term R3B1 environment within 14 days had a more pronounced promoting effect on anthocyanin content.

Under B light, Chl a contents consistently increased at all tested time points (**Figures 2G, H**). For instance, on day 7, Chl a content under B light reached 1.50 ± 0.12 mg g^-1^, in contrast to 1.10 ± 0.09 mg g^-1^ under R. By day 21, B light supported 1.58 ± 0.18 mg g^-1^ of Chl a, whereas R light showed only 1.30 ± 0.10 mg g^-1^, indicating inhibition of chlorophyll accumulation. Similarly, the Chl b content under B treatment also increased significantly at 21 days. White and R3B1 conditions exhibited moderate to high chlorophyll levels. Notably, R3B1 reached 1.80 ± 0.15 mg g^-1^ of Chl a on day 7 and increased further to 2.50 ± 0.25 mg g^-1^ on day 21.

### 3.3 Light environment significantly influences the OJIP transient response

OJIP transient kinetics can sensitively reflect the status of PSII electron transport, and different light qualities induce differential photochemical responses in seedlings. The shape of the OJIP transient response has been found to be highly sensitive to a variety of stress factors, such as excessive light, temperature, and drought (Xu et al., 2014). **Figure 3** shows the OJIP fluorescence kinetics on days 7, 14, and 21, respectively. Under four light quality conditions, the OJIP curves of all treatment groups exhibited the typical multiphasic OJIP rise, indicating that photosynthetic units remained at relatively normal levels, even under limited wavelengths (**Figure 3**). By calculating variable fluorescence Vt at each time point of the OJIP curve and normalizing to obtain ΔVt, the results reveal a marked elevation of the K phase and J phase under R light after 21 days of cultivation, which is also evident in the Vt curve (**Figure 3I**). An increase in Vk/Vj and ΔVk/ΔVj under R light suggests that the accumulation of PSII acceptors (QA and QB, which are the primary and secondary electron acceptors of PSII, respectively) and the plastoquinone (PQ) pool results in limited electron flow, often linked to constraints in downstream processes such as Rubisco activation (Borevitz et al., 2000). These findings are consistent with the observed reductions in leaf area, plant height, and biomass of ‘Harmony’ under R conditions, which may involve light-quality-mediated adjustments in stomatal function and development (Demotes-Mainard et al., 2016).

**Figure 3:**
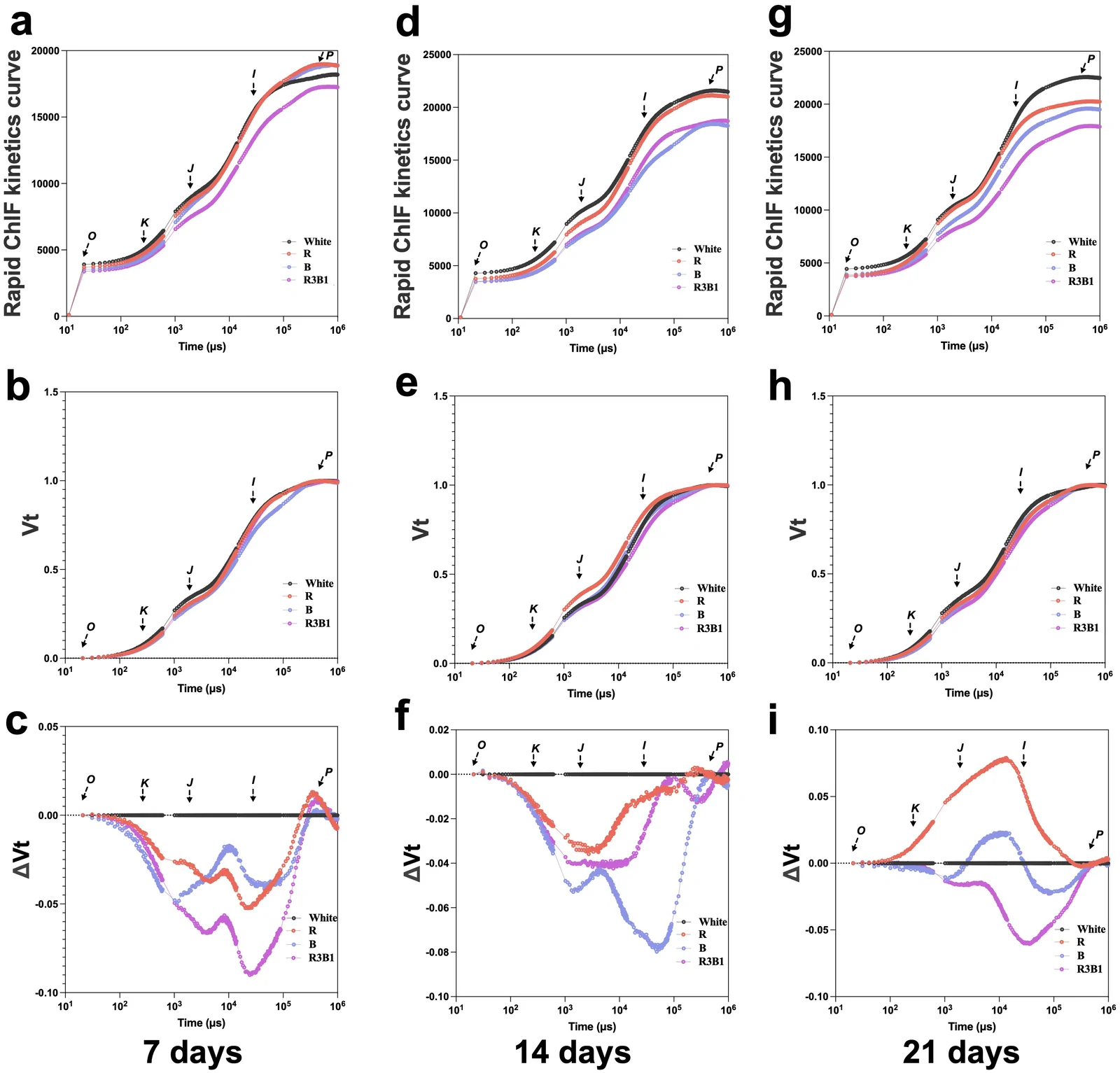
OJIP fluorescence transient curves **(A, D, G)**, variable fluorescence (Vt) **(B, E, H)**, and relative variable fluorescence (ΔVt) **(C, F, I)**. O, K, J, I, P represent time points at 20 μs, 300 μs, 2 ms, 30 ms, and the maximum fluorescence intensity, respectively. **(A–C)** Cultivation for 7 days; **(D–F)** cultivation for 14 days; **(G–I)** cultivation for 21 days.

### 3.4 TEM shows light environment leads to chloroplast development differences

Light quality significantly modulates chloroplast morphology, thylakoid structure and starch accumulation, thereby altering chloroplast developmental status. Under different light environments, chloroplast development varied significantly, as evidenced by the TEM (**Figure 4**). In the White and B light treatments, most chloroplasts were spindle-shaped or elliptical and aligned along the plasma membrane; these chloroplasts displayed clearly defined, densely stacked grana, facilitating effective capture of light energy and enhancing energy conversion efficiency during photosynthesis. As the proportion of R light increased, chloroplast number and the amount of both stromal and grana thylakoids declined substantially. Under R conditions, grana thylakoid development was incomplete or showed pronounced degradation, which reduced the energy conversion efficiency of the photosynthetic membranes and ultimately impaired overall photosynthetic performance. Additionally, transmission electron micrographs revealed varying increases in starch grains within chloroplasts across all treatments. Under R3B1 and R conditions in particular, starch grain content rose significantly, suggesting the onset of leaf senescence and an imbalance in carbohydrate metabolism (Tohge et al., 2005).

**Figure 4:**
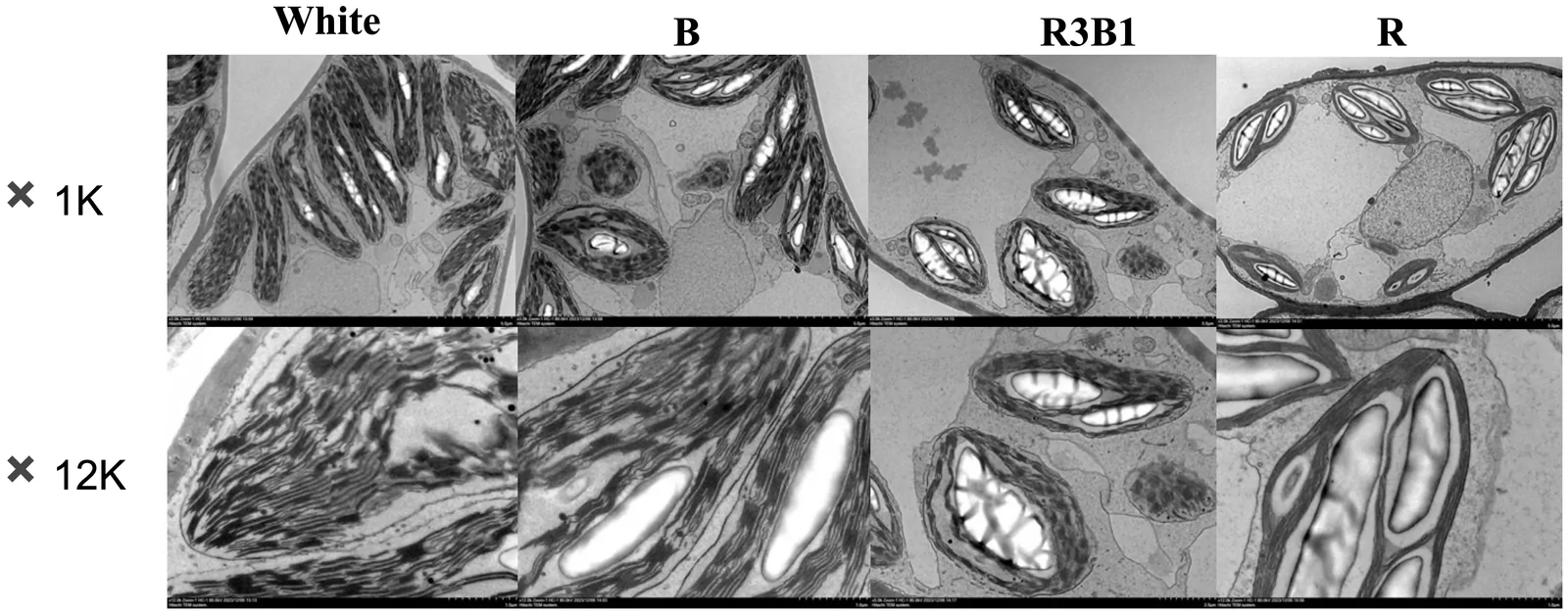
Transmission electron microscopy (TEM) images of chloroplast ultrastructure after 21 days of light treatment. 1K, magnification of 1,000×; 12K, magnification of 12,000×.

### 3.5 Light environments significantly affect endogenous hormone levels

Different light spectra significantly reshape endogenous hormone homeostasis, resulting in distinct accumulation patterns of key growth-related hormones. As shown in **Table 2**, the content of GA in the control group under White light was 60.57 ng g^−1^. Under B light treatment, the GA content decreased to its lowest level at 54.16 ng g^-1^, while under R3B1 treatment, it peaked at 78.38 ng g^−1^. Similarly, the content of BR was relatively low under B light treatment (15.03 ng g^-1^) and did not significantly differ from the control group under White light (14.28 ng g^-1^). In contrast, the highest BR content was observed under R light treatment (20.23 ng g^−1^), followed by R3B1 treatment (19.55 ng g^-1^). However, cytokinins (CTK) reached their highest concentration under B light treatment at 0.40 ng g^−1^, followed by R3B1 treatment at 0.36 ng g^−1^. These findings indicate that light quality significantly affects endogenous hormone levels, which may further influence plant growth and development.

**Table 2:** The content of endogenous hormones during a 21-day light cultivation period (ng g^-1^).

Compounds	White	B	R3B1	R
GA	60.57 ± 2.81^b^	54.16 ± 3.38^c^	78.38 ± 4.09^a^	64.77 ± 3.69^b^
BR	14.28 ± 1.06^b^	15.03 ± 0.68^b^	19.55 ± 0.63^a^	20.23 ± 1.45^a^
CTK	316.38± 17.00^c^	403.5± 22.17^a^	355.02 ± 19.13^b^	279.44 ± 24.24^d^

Values are the mean ± s.d. of three biological replicates. Different letters in each column represent significant differences using analysis of variance followed by Dunnett's T3 test.

### 3.6 RNA-seq data analysis

In order to identify genes exhibiting significant differential expression while accounting for biological variability, we performed principal component analysis (PCA) (**Figure 5A**) and calculated Spearman’s correlation coefficients (**Figure 5B**). The PCA results showed that the first two principal components explained 51.7% and 24.4% of the total variance, respectively, with each triplicate set clustering together. Four distinct distributions emerged, corresponding to the four light-quality treatments, revealing clear separation among the groups. Hierarchical clustering further reinforced these observations, as each treatment’s three replicates formed discrete clusters, indicating that variations in light quality profoundly affected the transcriptomic activity of ‘Harmony’.

**Figure 5:**
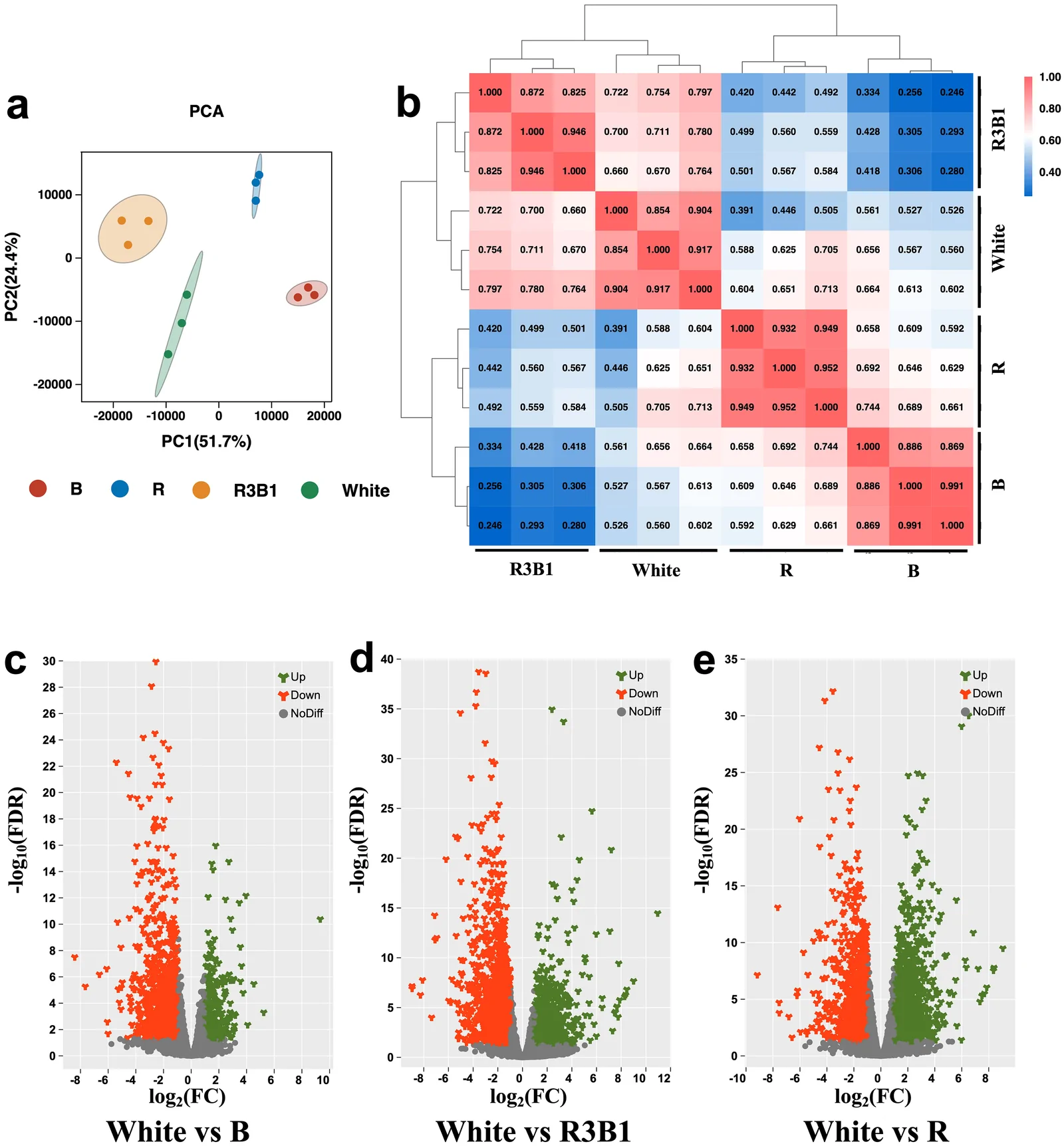
Principal component analysis (PCA) of sample reproducibility **(A)**, where axes represent different principal components, with percentages indicating their contributions to sample variation. Each point represents an individual sample, with different groups distinguished by colors and shapes; correlation coefficient (r) analysis **(B)** quantifying numerical values indicate the strength of correlation; volcano plots **(C–E)** visualize differentially expressed genes, log_2_(FC): Log-transformed fold-change in expression, -log_10_(FDR): Significance of differential.

Among the treatments, the R condition produced the highest number of DEGs, including 1,446 upregulated and 1,504 downregulated genes; this was followed by R3B1, with 825 upregulated and 1,504 downregulated genes. By contrast, B resulted in the fewest DEGs, comprising 458 upregulated and 986 downregulated genes (**Table 3**). The distribution of DEGs across treatments was visualized using volcano plots (**Figures 5C–E**), which underscore the substantial differences in transcriptional regulation under diverse light regimes. Overall, these findings suggest that ‘Harmony’ undergoes marked phenotypic and metabolic adaptations in response to shifts in light quality.

**Table 3:** Summary of the number of DEGs.

DEG Groups	DEG Number	Up-regulated	Down-regulated
White vs B	1,404	458	986
White vs R	2,950	1,446	1,504
White vs R3B1	2,422	825	1,597

### 3.7 Differentially expressed genes in photosynthetic pathways

Light quality differentially regulates the expression of photosynthesis-related genes, leading to distinct photosynthetic molecular regulation patterns under varied light conditions. Based on the expression analysis of photosynthesis pathway ko00195 under B light conditions (**Figure 6A**), we identified the corresponding DEGs (**Figure 6B**). Under the B-light condition, 15 DEGs were detected. Upregulated genes were predominantly implicated in energy production and conversion, as well as in the synthesis and assembly of cell walls and membranes. Fewer downregulated genes were similarly associated with energy production and conversion. Notably, under blue (B) light treatment, both PSI and PSII reaction centers—specifically core subunits PsaA/B/D/H and PsbA/C/S—were activated. This enhanced electron transport and improved the capture and transfer of light energy. Although *PsbS* does not directly participate in electron flow, it plays a vital role in non-photochemical quenching (NPQ) by modulating heat dissipation and preventing photodamage.

**Figure 6:**
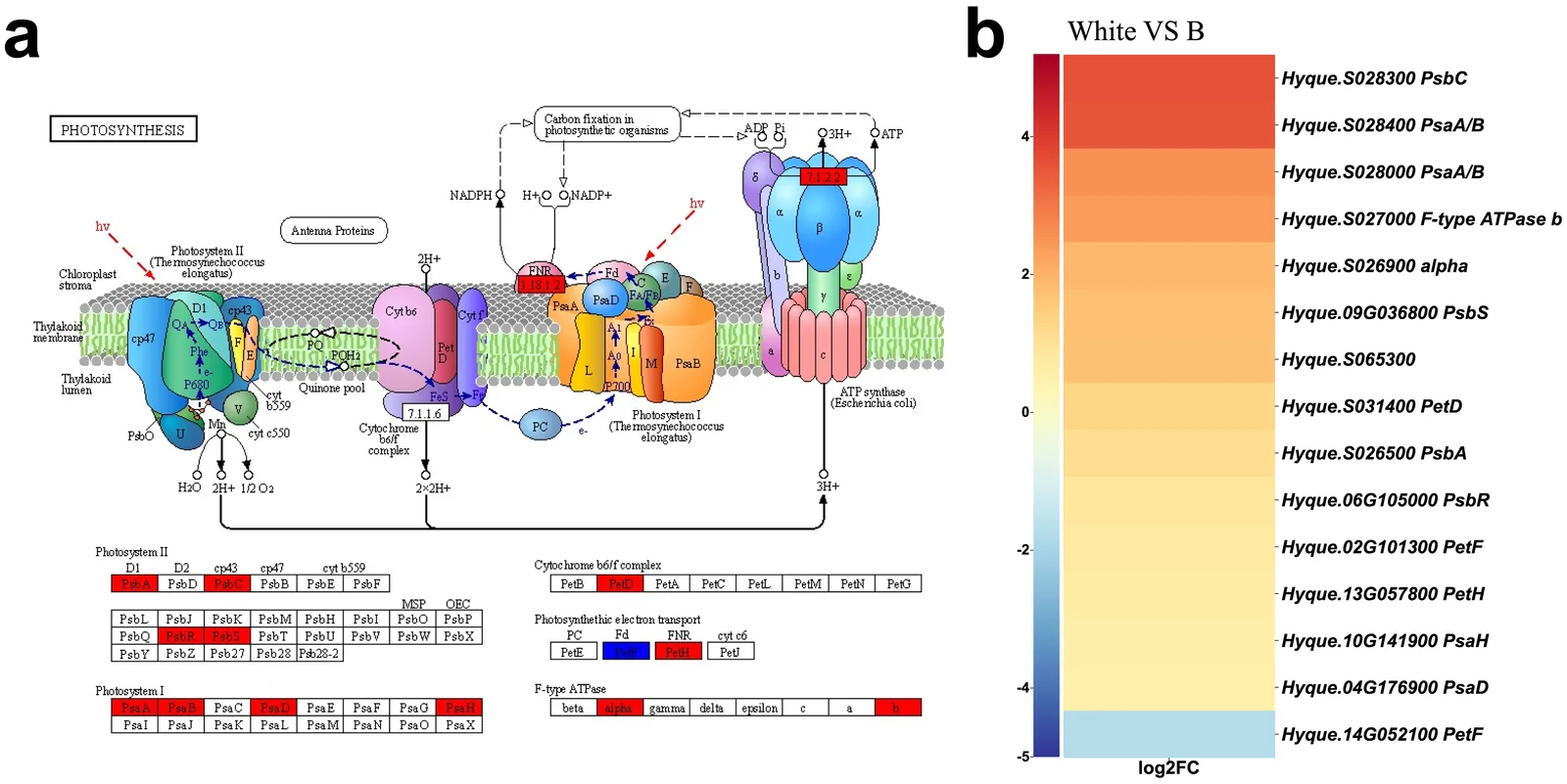
Pathway annotation diagram **(A)** of DEGs in the photosynthetic pathway for White vs B, and the differential expression fold changes of related genes **(B)**.

Under the R3B1 treatment, 68 photosynthesis-related DEGs were identified (**Figure 7A**), all of which were downregulated. These suppressed genes included the PSII oxygen-evolving complex, PSI reaction center, thylakoid membrane constituents, and cytochrome complex components (PetC/E/H/J). The PSII oxygen-evolving complex is a pivotal site in electron transfer during the light-dependent reactions, whereas PSI generates NADPH for carbon fixation. The marked inhibition of these genes indicates a pronounced repression of photosynthesis and electron transport under R3B1.

**Figure 7:**
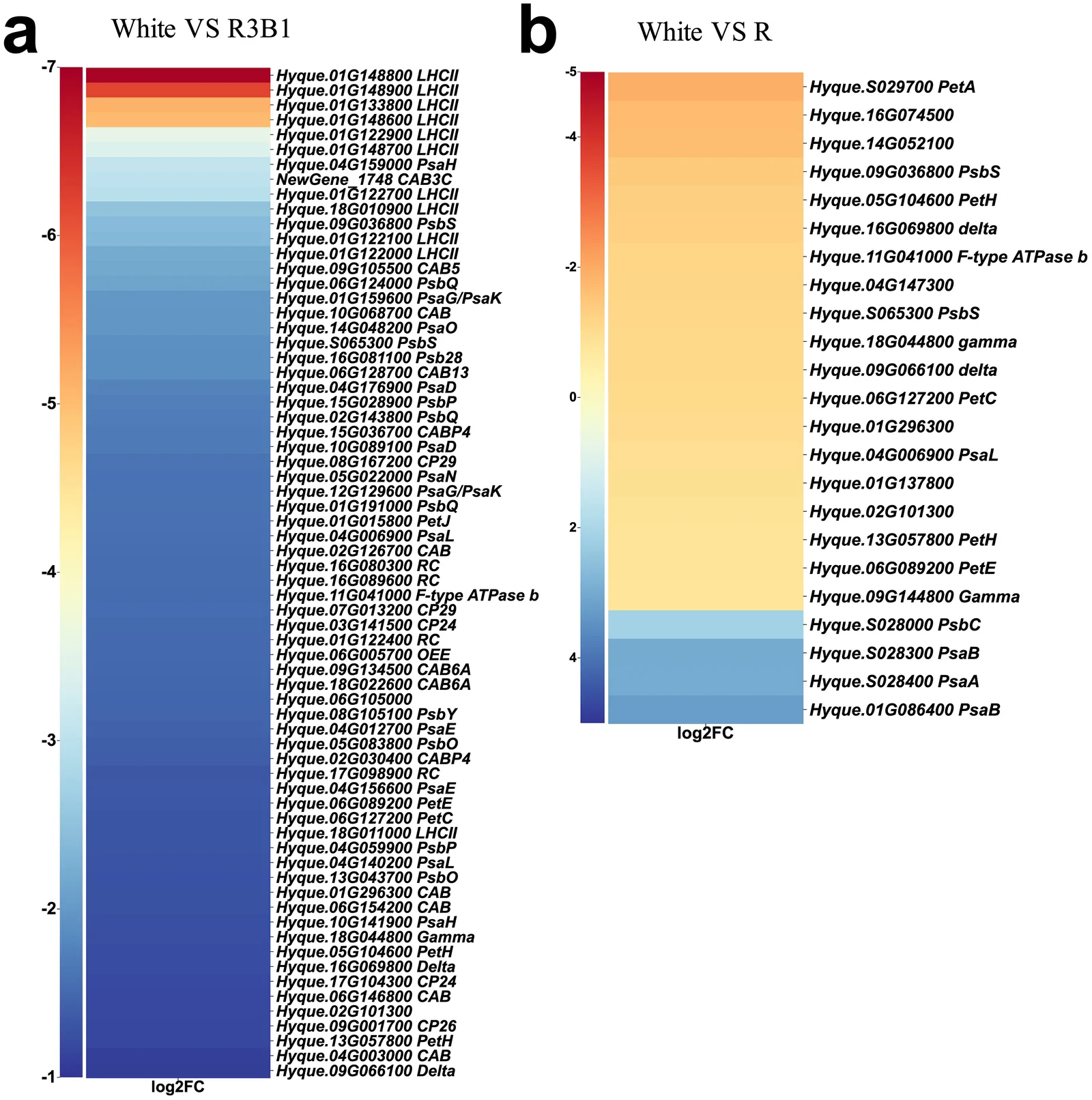
The differential expression fold changes of related genes in White vs. R3B1 **(A)**, and White vs. R **(B)**.

Finally, 23 photosynthesis-related DEGs were detected under R-light treatment (**Figure 7B**). While some genes—particularly those involved in cell wall formation and electron transport—were upregulated, a larger subset was downregulated, including genes critical for the structural composition of PSI and PSII, as well as chloroplast development (PetA/C/E/H). Consistent with these transcriptional changes, R light significantly reduced QY_Lss, NPQ_Lss, and ΦPSII, elevating ΦNO and suggesting that a greater proportion of absorbed light was dissipated as heat or fluorescence.

### 3.8 Differentially expressed genes in hormone metabolic pathways

KEGG enrichment analysis revealed that light quality induces widespread changes in the transcription of plant hormone signal transduction genes. In the hormone metabolic pathway, a larger number of DEGs were identified across the treatment groups. These DEGs were annotated based on their functional activity in the KEGG metabolic pathway, ko04075. Under the R3B1 treatment, 77 DEGs annotated to plant hormone–related pathways were identified (**Supplementary Figure S2A**), of which 33 were upregulated and 44 downregulated. The upregulated genes mapped to nuclear and membrane components, possessing DNA- and protein-binding activities; examples include *AUX1* and *GID1*. Meanwhile, the downregulated genes were involved in protein kinase activity and jasmonic acid (JA)/PR-1 defense responses, with *JAR1* and certain *DELLA* genes also exhibiting suppressed expression. Under the B light, 39 DEGs were annotated to plant hormone signal transduction pathways in the KEGG database (**Supplementary Figure S2**). Among these, 5 were upregulated and 34 were downregulated. The upregulated genes primarily encode protein kinases, ATP-binding proteins, and protein Ser/Thr phosphatases, and are linked to “nuclear function,” “DNA-templated transcriptional regulation,” and “auxin-activated signaling pathways.” Notably, genes related to *JAR1*, *ARF*, and *AUX/IAA* were found among the upregulated group. In contrast, many of the downregulated genes are associated with ABA-related signaling pathways and phosphatase inhibitory activity; *BRI* and *DELLA* genes were also suppressed.

Finally, under R treatment, the pathway analysis related to plant hormone metabolism or signal transduction is shown in **Figure 8A**, which contained the highest number of corresponding DEGs, with a total of 94 DEGs comprising 49 upregulated and 45 downregulated genes. Among these, the 23 most significantly DEGs are displayed in **Figure 8B**. The upregulated genes were largely clustered in auxin and BR pathways; in the cytokinin pathway, *CRE1* and *ARR-A* displayed both increased and decreased expression, and *DELLA* genes in the GA pathway exhibited diverse patterns. The downregulated genes primarily function in phosphate sensing, DNA-binding transcription factor activity, and stress responses to external stimuli; notably, some *CRE1* genes were strongly suppressed.

**Figure 8:**
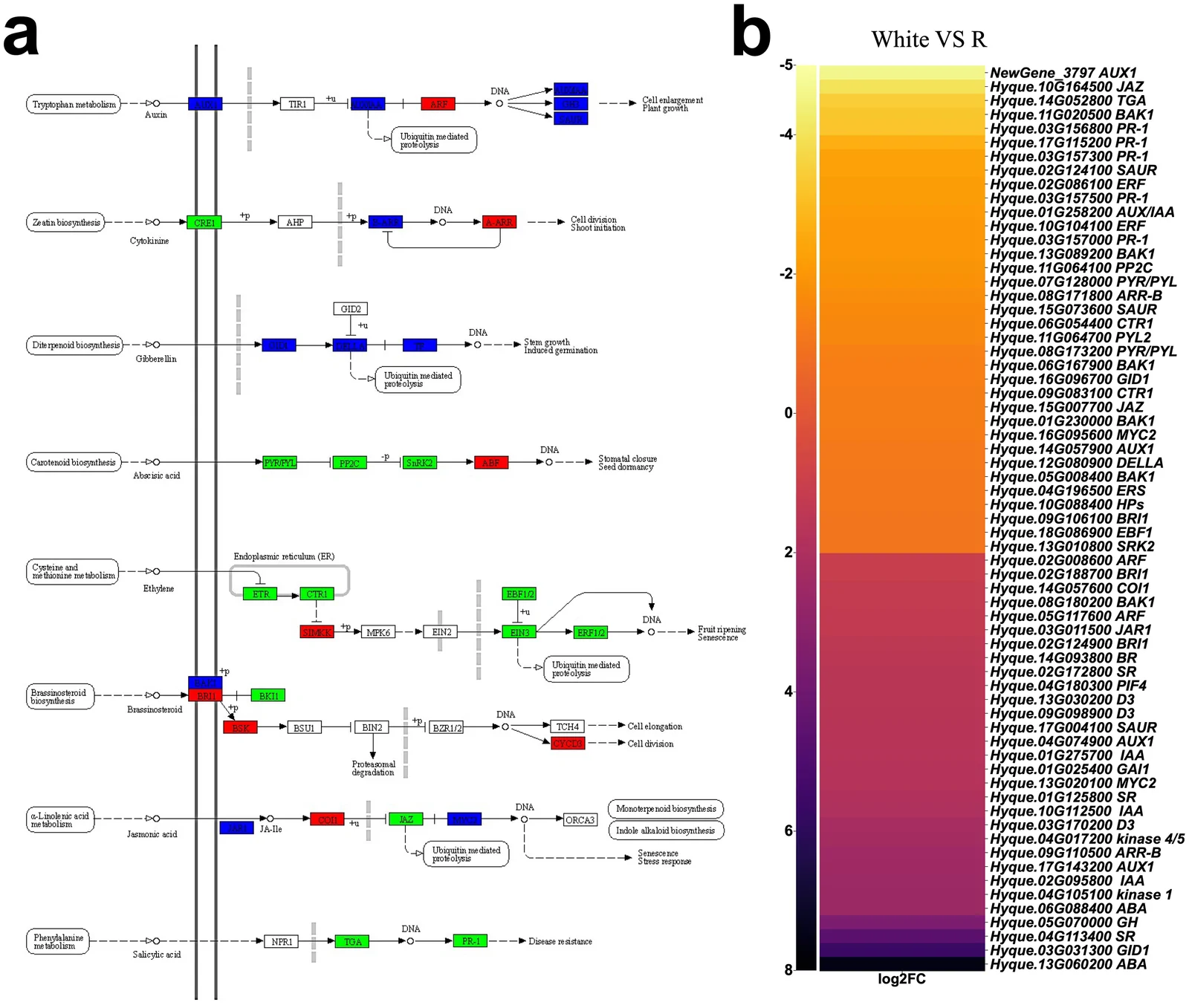
Pathway annotation diagram **(A)** of DEGs in the plant hormone signaling pathway for White vs R, and the differential expression fold changes of related genes **(B)**.

## 4 Discussion

Different light spectra differentially regulate chlorophyll synthesis and anthocyanin accumulation, thereby affecting leaf pigment characteristics of hydrangea seedlings. This study assessed chlorophyll and anthocyanin content in ‘Harmony’ seedlings over a 21-day light treatment period. The results demonstrated that B light significantly promoted chlorophyll synthesis, whereas R inhibited chlorophyll accumulation in the later stages of cultivation. In contrast, anthocyanin content increased significantly on days 7 and 14, particularly under B and R3B1 treatments, while anthocyanin levels in the R treatment declined markedly by day 21. These findings are consistent with previous research indicating that B light enhances anthocyanin concentration by regulating plant metabolic pathways, thereby promoting plant growth (Stutte et al., 2009) and increasing anthocyanin levels in strawberries under postharvest storage conditions (Zhang et al., 2025). The present study also observed a significant increase in anthocyanin content under B treatment as early as day 7. As cultivation progressed, the advantage of R3B1 in anthocyanin accumulation became more pronounced, suggesting that both B and R3B1 positively influence anthocyanin synthesis at different developmental stages.

The promotion of chlorophyll biosynthesis by B light is likely associated with the activation of key photosynthetic and photomorphogenetic pathways, possibly mediated by cryptochromes and other B light receptor (Jiang et al., 2023). Conversely, the inhibitory effect of R light on chlorophyll accumulation in the later stages may be linked to phytochrome-mediated responses (Gao et al., 2018), which influence resource allocation, photosynthetic efficiency, or gene regulation.

Regarding anthocyanin accumulation, B light exhibited an enhancement effect as early as day 7, aligning with previous studies that reported B light upregulates gene expression and enzymatic activity in the flavonoid biosynthetic pathway, thereby promoting pigment deposition and potentially enhancing stress resistance (Zhou et al., 2024). Moreover, *MYB75* and *MYB90* are key transcriptional regulators in the phenylpropanoid metabolic pathway. Overexpression of *MYB75* has been shown to cause a significant accumulation of anthocyanins, altering the plant’s coloration phenotype. Additionally, high expression of *MYB75* often induces an upregulation of *MYB90*, which, when co-induced by light and sugar signaling, can further promote anthocyanin biosynthesis (Fan et al., 2024; Zuo et al., 2026). In this study, anthocyanin content in B and R3B1 treatments continuously increased over time, suggesting a close correlation with the transcriptional upregulation of *MYB75* and *MYB90*. Conversely, the diminished effect of R treatment on anthocyanin synthesis in the later stages may be attributed to insufficient induction of these transcription factors or the involvement of inhibitory regulators.

Although B treatment alone produced notable effects during the early phase, the R3B1 maintained higher anthocyanin levels over a prolonged cultivation period. This synergistic effect may arise from the complementary interactions between red and blue light in photoreceptor activation or metabolic regulation, warranting further studies to elucidate its molecular basis.

Light quality alterations significantly affect PSII electron transport efficiency, and red light induces obvious photoinhibition at the seedling late growth stage. Measurements of OJIP fluorescence transient curves were taken on days 7, 14, and 21 (**Figure 3**). Although all treatments exhibited the characteristic multiphasic OJIP rise, the R-light treatment displayed notable elevations in the K and J phases at day 21 (**Figure 3I**). In general, the K phase and J phase of the OJIP curve are closely tied to specific electron transport processes on the donor and acceptor sides of PSII, respectively (Xu et al., 2014). An elevated K phase often indicates a transient functional impairment in the oxygen-evolving complex (OEC) or water-oxidation site, while a pronounced J phase rise is linked to the accumulation of reduced QA^−^ and QB^−^ and an electron “traffic jam” in the plastoquinone (PQ) pool, thereby hindering normal electron flow (Borevitz et al., 2000; Arshad et al., 2026). In this study, both Vk/Vj and ΔVk/ΔVj increased in the R treatment group, corroborating the presence of blockages at both the donor and acceptor sides of PSII and ultimately restricting electron flux, which diminished photosynthetic performance.

Consistent with earlier findings, ‘Harmony’ seedlings under R light exhibited reduced leaf area, plant height, and biomass, likely stemming from the inhibited electron transport in PSII and the consequent reduction in energy and assimilates needed for growth. Previous reports have highlighted the sensitivity of OJIP transient curves to a wide range of environmental stressors, such as excessive light, extreme temperatures, or drought (Xu et al., 2014; Wang et al., 2022; Kishi et al., 2026). In the present study, the distinct elevations in the K and J phases under R light suggest that functional impairments on the donor and acceptor sides of PSII—or excess energy within the reaction center—led to these characteristic OJIP fluctuations, ultimately manifesting in a suite of morphological and physiological changes.

Transcriptomic analysis further revealed distinct regulatory mechanisms of B, R, and R3B1 light on photosynthesis and chloroplast development at the molecular level. Transcriptome sequencing revealed that, under B-light conditions, upregulated genes notably promoted photosynthesis and biochemical processes related to energy capture. In particular, the activation of key enzymes in the PSI and PSII reaction centers—especially those associated with the PSII oxygen-evolving complex and the electron transport chain—ensured efficient conversion of light energy into chemical energy (ATP and NADPH), thereby bolstering photosynthetic performance (Ye et al., 2024). Moreover, elevated *PsbS* expression proved critical for the regulation of non-photochemical quenching (NPQ) (Chen et al., 2024). An increase in NPQ reflects enhanced thermal dissipation (Ramakers et al., 2026), safeguarding the photosynthetic apparatus from light-induced damage and improving stress tolerance. The upregulation of these genes is consistent with the research group’s previous findings, which showed an increase in NPQ_Lss and ΦPSII (Su et al., 2024), indicating that B light enhances photosynthesis and photoprotection. Overall, this evidence indicates that B light not only augments photosynthetic efficiency through transcriptional regulation but also fortifies photoprotective mechanisms, ultimately advancing the morphogenesis.

Under the R3B1 condition, a broad array of photosynthesis-related genes showed significant downregulation, most notably those encoding key components of PSI and PSII reaction centers. In particular, suppression of the PSII oxygen-evolving complex directly restricted the release of electrons during the light-dependent reactions, thereby impeding the normal progression of the photosynthetic electron transport chain (Shan et al., 2024). Concurrently, the reduced capacity for PSI-mediated electron transport and NADPH production further weakened carbon fixation (You et al., 2024). This inhibition of core electron transport steps not only curtailed the efficient use of absorbed light energy but also potentially affected subsequent energy and metabolic pathways.

Moreover, the downregulation of *PetC*/*E*/*H*/*J* impaired chloroplast structural development (Blaby-Haas, 2021; Liu et al., 2026), as evidenced by fewer chloroplasts and decreased numbers of stromal thylakoids, which were also incomplete or exhibited pronounced degradation under R3B1—likely attributable to the significantly reduced expression of these genes. This compromised chloroplast structure and function further limited photosynthetic efficiency. Taken together, R3B1 imposes a multilayered inhibition of photosynthesis, involving both direct restrictions on electron transport and disruptions to chloroplast development, ultimately exerting a pronounced impact on plant morphogenesis.

Under R light treatment, genes involved in cell wall formation and electron transport were upregulated. However, genes encoding structural components of PSI and PSII, as well as those tied to chloroplast formation, showed widespread repression, resulting in a marked decline in photosynthetic efficiency (Si et al., 2024). This repression diminished PSII’s capacity for light absorption and oxygen evolution, and simultaneously hindered electron reception and NADPH production in PSI (Pi et al., 2019). Consistent with these molecular observations, R light significantly reduced the parameters QY_Lss, NPQ_Lss, and ΦPSII, while elevating ΦNO, signifying that more absorbed light energy was passively dissipated as heat or fluorescence (Su et al., 2024). The diminished utilization of light energy indicates that R light, by inhibiting chloroplast development, electron transport, and light signal transduction, substantially weakens the photosynthetic capability of ‘Harmony’ and its ability to adapt to light environments. Consequently, red light exerts a negative effect on photosynthesis and further inhibits the subsequent growth and development of *H. quercifolia* seedlings.

Light spectra remodel endogenous hormone signaling networks, thereby mediating seedling growth differences under varied light environments. In the hormone metabolic pathways, B-light conditions significantly upregulated genes integral to auxin (Auxins) and JA signaling. The elevated expression of ARF (Auxin Response Factor) transcription factors suggests an intensified auxin response pathway, which supports cell elongation and division (Cancé et al., 2022). Likewise, upregulation of JAR1 (jasmonic acid amino acid transferase) and related genes indicates the activation of the JA signaling pathway, potentially playing a pivotal role in governing plant growth and reinforcing stress tolerance mechanisms (Mahmud et al., 2022). By contrast, key genes such as *BRI* and *DELLA*, involved in BR and GA pathways, were downregulated, implying that while B light stimulates auxin and JA signals, it concurrently suppresses BR and GA pathways. Measurements of endogenous hormone levels further corroborated this finding: under B-light conditions, the levels of BR and GA in ‘Harmony’ were substantially lower than those in other treatments, suggesting that the balance among multiple hormone signals under B light ultimately strengthens morphological development.

Under R3B1 conditions, hormone signaling pathways exhibit a multi-tiered and intricately interconnected regulatory landscape. The upregulation of *AUX1* and *GID1* bolsters auxin and GA signaling, thereby partially driving cell division and elongation (Hirano et al., 2010; Zheng et al., 2024). However, the pronounced suppression of *JAR1* and *PR-1* indicates that R3B1 constrains both JA–mediated processes and pathogen-related protein expression, curtailing certain defense and growth regulatory functions. Although the downregulation of *ARR-B* suggests inhibited cytokinin transport (Zhao et al., 2024), measurements of endogenous hormone levels hint at a moderate increase in cytokinin content, implying additional genetic factors may be compensating for this regulatory gap. Meanwhile, the partial repression of some *DELLA* genes points to a possible activation of GA signaling (Shi et al., 2024), though fluctuations in other inhibitors and responsive elements did not align perfectly. These observations underscore that R3B1 neither promotes nor suppresses hormone pathways in a unidirectional manner; rather, it orchestrates a complex interplay among multiple hormonal signals.

Under R light treatment, the upregulation of selected genes in auxin and BR pathways provides some support for cell elongation and differentiation (Wu et al., 2024). Nevertheless, numerous genes tied to CTK and GA pathways exhibit downregulation or unstable expression. Notably, the significant repression of *CRE1* inhibits cytokinin signaling (Zhao et al., 2024), aligning with measurements of endogenous cytokinin levels. Expression of *DELLA* family genes in the GA pathway shows both up- and downregulation, highlighting the diverse and feedback-rich nature of GA regulation under R light. Taken together with the declining expression of external stimulus–responsive genes. Collectively, these results indicate that R light can activate certain growth-related hormone pathways, which partially sustains the physiological activity of *H. quercifolia* seedlings. However, the predominant inhibition of CTK and GA pathways disrupts normal seedling growth and development, further demonstrating that single monochromatic R light is unsuitable for the seedling cultivation of hydrangea.

## 5 Conclusion

Treatment with B light enhanced light capture and energy conversion efficiency, improving photosynthetic performance. Chlorophyll fluorescence parameters indicated that under B light treatment, a greater proportion of light energy was utilized for photosynthesis, with significant increases in ΦPSII and NPQ_Lss, which facilitated effective photoprotection. In contrast, R light treatment reduced ΦPSII and light energy utilization efficiency, causing a larger proportion of absorbed light energy to be dissipated as heat and fluorescence and consequently suppressing seedling growth. Structurally, B light treatment maintained intact chloroplast morphology and stable thylakoid structure, thereby preserving normal light energy absorption, transport, and conversion capacities. Conversely, R light treatment reduced chloroplast number, induced underdeveloped grana thylakoids, and decreased photosynthetic efficiency. Excessive starch accumulation in R-treated chloroplasts implied disrupted carbon metabolism homeostasis, which may contribute to leaf senescence. Transcriptome analysis showed that B light treatment upregulated core genes associated with PSI and PSII, supporting photosystem stability and photoprotection. In comparison, both R3B1 and R light treatments repressed genes involved in chloroplast development and electron transport, impairing light energy capture and utilization. In terms of hormone regulation, B light treatment decreased endogenous BR and GA contents and downregulated the defense-related gene *PR-1*. R3B1 light treatment activated GA and ethylene signaling pathways, which may accelerate seedling maturation and senescence. Under monochromatic R light, CTK biosynthesis was inhibited while BR signaling was enhanced, jointly altering seedling growth and developmental patterns.

## Data Availability

The datasets presented in this study can be found in online repositories. The names of the repository/repositories and accession number(s) can be found in the article/**Supplementary Material**.

## Supplementary material

Supplementary Figure 1 RNA gel electrophoresis analysis of ‘Harmony’ seedlings cultured under different light conditions for 21 days.

Supplementary Figure 2 Differential expression fold changes of related genes involved in the plant hormone signaling pathway in White vs. B and White vs. R3B1.
