# Supplementary material for: Blue light optimizes photosynthetic physiology and photoprotective molecular mechanisms of Hydrangea quercifolia seedlings
Source: Front Plant Sci. 2026 Oct 13;17:1951910. doi: 10.3389/fpls.2026.1951910 (PMC13710404; doi:10.3389/fpls.2026.1951910)
Supplement: Supplementary file 1 [file DataSheet1.docx]

Supplementary Material

# 1 Supplementary Figures and Tables

## 1.1 Supplementary Figures

**Fig. S1**. RNA gel electrophoresis analysis of 'Harmony' seedlings cultured under different light conditions for 21 days.

**Fig. S2**. Differential expression fold changes of related genes involved in the plant hormone signaling pathway in White vs. B and White vs. R3B1
